# FFPE-Based NGS Approaches into Clinical Practice: The Limits of Glory from a Pathologist Viewpoint

**DOI:** 10.3390/jpm12050750

**Published:** 2022-05-05

**Authors:** Filippo Cappello, Valentina Angerilli, Giada Munari, Carlotta Ceccon, Marianna Sabbadin, Fabio Pagni, Nicola Fusco, Umberto Malapelle, Matteo Fassan

**Affiliations:** 1 Department of Medicine (DIMED), University of Padua, 35128 Padua, Italy; flppcappello@gmail.com (F.C.); valentina.angerilli@gmail.com (V.A.); carlotta.ceccon@unipd.it (C.C.); 2 Veneto Institute of Oncology, Istituto di Ricovero e Cura a Carattere Scientifico (IRCCS), 35128 Padua, Italy; giadamunari@gmail.com (G.M.); mariannasabbadin310@gmail.com (M.S.); 3 Department of Medicine and Surgery, Pathology, University Milan Bicocca, 20900 Milan, Italy; fabio.pagni@unimib.it; 4 Department of Oncology and Hemato-Oncology, University of Milan, 20122 Milan, Italy; nicola.fusco@unimi.it; 5 Division of Pathology, IEO, European Institute of Oncology IRCCS, 20141 Milan, Italy; 6 Department of Public Health, University of Naples Federico II, 80131 Naples, Italy; umbertomalapelle@gmail.com

**Keywords:** NGS, diagnostics, precision medicine, biomarkers

## Abstract

The introduction of next-generation sequencing (NGS) in the molecular diagnostic armamentarium is deeply changing pathology practice and laboratory frameworks. NGS allows for the comprehensive molecular characterization of neoplasms, in order to provide the best treatment to oncologic patients. On the other hand, NGS raises technical issues and poses several challenges in terms of education, infrastructures and costs. The aim of this review is to give an overview of the main NGS sequencing platforms that can be used in current molecular diagnostics and gain insights into the clinical applications of NGS in precision oncology. Hence, we also focus on the preanalytical, analytical and interpretative issues raised by the incorporation of NGS in routine pathology diagnostics.

## 1. Introduction

Next-Generation Sequencing (NGS) is a technology that allows the sequencing of large genomic regions (or even of the entire genome) in a short amount of time and with an affordable cost, through the sequencing of millions of DNA fragments at the same time. NGS is able to detect genomic alterations, such as base substitutions, insertions, deletions, copy number alterations and gene rearrangements, and it can also be used to detect alterations of gene expression and epigenetic variations [1].

NGS has recently moved into clinical practice, allowing the detection of alterations with diagnostic, prognostic and predictive value. In this context, pathologists have assumed a pivotal role, becoming central figures in therapeutic decisions, the identification of new biomarkers and translation of biomarker discovery into clinical practice. This new role requires the knowledge of molecular technologies, their potential and their limits.

Formalin-fixed paraffin-embedded (FFPE) tissues are the primary resource for tumor molecular characterization. However, nucleic acids extracted from FFPE tissues are fragmented and chemically altered, making them challenging to use in molecular diagnostics. Since the first reports regarding the accuracy of the FFPE-derived DNA for NGS-based analyses were published [2], “FFPE-friendly” approaches have been implemented within the NGS pipeline [3].

In the present review, we briefly describe the most used sequencing platforms, the role of NGS in clinical practice according to the most recent recommendations and its preanalytical and analytical issues. We also assess the role of RNA-based NGS and the issues related to the interpretation of sequencing data.

## 2. Sequencing Platforms

In 1977, the first method for sequencing DNA, known as Sanger sequencing, was described. This method is now considered the first-generation sequencing technology [4,5]. By using Sanger sequencing, only a single sequencing reaction could be read at a time, and this implies that this method is of limited throughput, in addition to having a high cost. Between 2004 and 2005, next-generation sequencing was developed in order to reduce the time and cost of genome sequencing [6]. The first NGS technologies to be introduced (referred to as second-generation sequencing) generally required DNA fragmentation, DNA end-repair, adapter ligation, surface attachment and in situ amplification. Second-generation sequencing is based on the parallel sequencing of millions of relatively short DNA fragments. Therefore, when sequencing long stretches of DNA, the short reads relative to each fragment must be put together, and this operation can be challenging, especially in the presence of structural variations and low-complexity regions [7]. Instead, third-generation sequencing can achieve read lengths of more than 10 kb, and is able to directly target unfragmented DNA molecules in real time. These long-read technologies can overcome issues encountered with second-generation systems, such as the sequencing of repetitive regions, the analysis of structural variants or the study of the overall chromosome structure [8]. However, the nucleic acids extracted from FFPE samples are usually fragmented and of relatively poor quality, and they do not allow for long reads; therefore, even if FFPE samples are theoretically compatible with long-read technologies, other sources of DNA or RNA, such as fresh frozen tissues, are required to take advantage of third-generation sequencing [9]. Moreover, compared to short-read systems, third generation sequencing in its beginnings guaranteed less read accuracy, but this aspect is progressively improving [10].

The most common sequencing platforms available on the market, including both short-read (Ion Torrent, Illumina and GeneReader) and long-read sequencing platforms (Pacific Biosciences and Oxford Nanopore Technology) are briefly reviewed below.

### 2.1. Ion Torrent

On the Ion Torrent platform, the DNA fragments are attached to microbeads and are then amplified through an emulsion PCR [11]. After this process, millions of beads with millions of different fragments are generated. The beads are then deposited into a chip, in such a way that each microwell in the chip contains a single bead. After that, the chip is flooded with one of the four nucleotides. When a nucleotide is incorporated into the growing strand of DNA, a hydrogen ion is released, modifying the pH of the solution contained in the microwell. The pH change is detected by a complementary metal-oxide semiconductor (CMOS) pH sensor, which converts pH modifications into voltage changes. The voltage change is then recorded, indicating the incorporation of the specific nucleotide added into the solution [12,13]. Ion Torrent allows fast sequencing, with read lengths between 200 and 600 bp. The main limit of this platform is the difficulty of sequencing regions with mononucleotide repeats, because, with the incorporation of multiple identical nucleotides, the linearity of the voltage change measurement may be lost. This can lead to an erroneous detection of insertions or deletions [14]. Different Ion Torrent platforms include the IonGeneStudio 5S, Genexus and Ion PGM Dx instruments.

### 2.2. Illumina

On the Illumina platform, DNA fragments are anchored to the surface of flow cell channels, and bridge PCR is used to amplify the DNA fragments. As Ion Torrent technology, Illumina uses a “sequencing by synthesis” approach [15]. In this case, during the sequencing process, fluorescently tagged nucleotides compete for the incorporation into the growing strand. After the addition of each nucleotide, the fluorescent label is excited by a light source, and the characteristic fluorescent signal associated with each specific nucleotide is emitted. The light signal is recorded through a high-resolution electronic camera and used for determining the DNA sequence [6]. The maximum read length varies according to the instrument in use. For example, the Illumina MiSeq instrument favors fragment sizes of 350–500 bp, but longer fragments can be read. Illumina technology is the most accurate NGS technology on the market, with an error rate of approximately 0.1% [7]. The main disadvantage is the relatively long sequencing run time. Different Illumina platforms include sequencing platforms (iSeq 100, Miseq, MiniSeq, NextSeq 550, NextSeq 100, NextSeq 2000, HiSeq 2500, HiSeq 3000, HiSeq 4000, NovaSeq 5000 and NovaSeq 6000), as well as in vitro diagnostic instruments (MiseqDx and NextSeq 550 Dx). These platforms have different performance characteristics; the maximum reads per run and maximum outputs range from 4 million and 1.2 Gb, respectively, for iSeq100 to 20 billion and 6000 Gb for Novaseq 6000. While benchtop sequencers, such as the iSeq, Miseq and MiniSeq platforms, find application in targeted gene sequencing and targeted gene expression profiling, production-scale sequencers such as NextSeq, NovaSeq and HiSeq platforms are also used to perform WES, WGS, methylation and single-cell sequencing.

### 2.3. GeneReader

On the GeneReader platform, after clonal amplification through an emulsion PCR, DNA fragments are attached to the flow cell. During the “sequencing by synthesis” process, modified nucleotides with a removable fluorescent dye and an end cap compete for the incorporation into the growing strand. As in the case of Illumina, the array is scanned by a high-resolution camera and the fluorescent signal emitted by each nucleotide after its incorporation is measured and recorded [16]. GeneReader has demonstrated a high accuracy in mutation calling, reaching high concordance with other NGS platforms and with Sanger sequencing [16,17].

### 2.4. Pacific Biosciences

Pacific Biosciences (PacBio) sequencing is a third-generation sequencing technology. On this platform, the DNA to be sequenced is in the form of a single-stranded circular DNA (called the SMRTbell template). This template is generated by ligating hairpin adaptors to the extremities of a double stranded DNA fragment. The SMRTbell templates are placed into a chip called the SMRT Cell. This chip contains millions of microwells, referred to as zero-mode waveguides (ZMWs), each containing an individual DNA polymerase. A single molecule of circular DNA is immobilized in a single ZMW and the polymerase starts incorporating fluorescently labeled nucleotides. The binding of a nucleotide to the polymerase generates a light signal, which is used for determining the DNA sequence [18,19]. This method does not require DNA amplification prior to sequencing; thus, preventing errors due to PCR amplification. Through the PacBio Iso-Seq method, it is also possible to sequence full-length cDNA generated by transcripts, using PacBio SMRT sequencing technology. Through PacBio technology, accurate long reads are possible, with a maximum read length of 300 kb. This method obtained precision and recall rates of at least 99.91% for single-nucleotide variants (SNVs), 95.98% for insertions and deletions of <50 bp and 95.99% for structural variants [20]. The majority of sequencing errors is due to indels, and a small amount is due to miscalls. The error rate can be reduced by multiple sequencing runs [21].

### 2.5. Oxford Nanopore Technology

Oxford nanopore technology (ONT) is a third-generation sequencing method that is capable of reads longer than 1 Mb [22]. ONT uses protein nanopores (staphylococcal α-hemolysin) embedded in a synthetic membrane and bathed in a solution containing electrolytes. The ions present in the solution pass through the nanopores, generating an ionic current. During the sequencing process, a single-stranded DNA or RNA molecule moves through the nanopore. The passage of each nucleotide through the pore causes a specific disruption in the ion current, which is measured and used for the determination of the nucleic acid sequence. As PacBio technology, ONT does not require amplification prior to sequencing; moreover, it can also use native RNA, avoiding the passage of cDNA synthesis. With this technology, most reads, both standard long and ultra-long, achieve an 87–98% accuracy [23]. The relatively low accuracy limits the utility of this technology for single-nucleotide variant calling [24].

Table 1 summarizes the main technical characteristics of different NGS platforms: Illumina MiSeq, Illumina HiSeq 2000, Ion Torrent PGM, PacBio SMRT and Oxford Nanopore MinION.

## 3. NGS in Clinical Practice

NGS can be used to determine the sequence of specific genes, or parts of them (targeted panels), to sequence all the coding regions in the genome (whole-exome sequencing, WES) or to sequence the entire genome, including intronic and intergenic regions (whole-genome sequencing, WGS) [25]. NGS allows for comprehensive genomic profiling (CGP), which consists of evaluating hundreds of genes as well as genomic signatures (such as microsatellite instability and the tumor mutation burden) in order to find clinically relevant molecular alterations. To date, an opening challenge point is represented by the accuracy level shown by different commercially available NGS platforms. Conventionally, the accuracy level should be optimized at >99.0% values [26]. Standardized guidelines highlight that a minimum of 500× and 1000× coverage depth are required for tissue and liquid biopsy analyses, respectively. Several technical parameters impact on the successful rate of NGS analysis: the number of tested samples in each NGS run, number of simultaneous tested genes (reference range of NGS panel), amount of starting DNA/RNA concentration and the size of support used for the sequencing phase [27].

Currently, WES and WGS are utilized for research purposes only. Instead, targeted panels find increasing applications in clinical practice because they are usually less expensive and time consuming than exome or genome sequencing, and they provide easier to interpret data on clinically relevant genes [28].

Other technologies currently in use in molecular diagnostics include Sanger sequencing, quantitative real-time PCR (qRT-PCR), reverse transcriptase PCR (RT-PCR), microarray platforms and fluorescent/chromogenic in situ hybridization (FISH/CISH). Sanger sequencing is a low-cost and widespread technology, but has a high turn-around time and is currently used for small-scale projects and for the validation of deep sequencing results. qRT-PCR and RT-PCR are characterized by a great diagnostic sensitivity, a fast turn-around-time and low-cost machinery; however, alteration-specific primers are required. Microarray technologies and FISH/CISH are hybridization-based technologies. Microarray can detect the expression of thousands of genes from a sample, but it can be used only for the analysis of predefined sequences, and hybridization can potentially be nonspecific. FISH/CISH can detect and localize a specific DNA sequence alteration, but requires expertise and is time consuming. To date, qRT-PCR and microarray technologies are considered the gold standard to measure the gene expression level. In this scenario, several methodological limitations drastically impact on the accuracy value among different technical approaches. In this regard, RNAseq is considered an emerging but robust and reproducible assay for the evaluation of expression gene levels, as demonstrated by Corchete et al. [29].

In 2020, the European Society of Medical Oncology (ESMO) was the first scientific society to publish recommendations on the use of NGS for patients with advanced cancers [30]. Based on the available evidence, the ESMO recommends the routine use of NGS on a series of tumors: nonsquamous nonsmall cell lung cancer (NSCLC), prostate adenocarcinoma, ovarian carcinoma and cholangiocarcinoma.

In nonsquamous NSCLC, tumor multigene NGS is recommended for the identification of genomic alterations with a predictive value that has been established in clinical trials [31]. Among these alterations, *EGFR* in-frame activating mutations in exon 19 and point-activating mutations in exon 21 (L858R) predict the efficacy of EGFR tyrosine kinase inhibitors (TKIs), such as erlotinib and gefitinib; mutations in exon 20, on the other hand, are associated with the acquisition of drug resistance [32,33]. *ALK* fusions are associated with patient response to anaplastic lymphoma kinase (ALK) inhibitors, such as crizotinib [34,35]. Other predictive biomarkers in nonsquamous NSCLC are the *MET* exon 14 skipping mutations [36,37], *BRAF* V600E mutation [38], *ROS1* fusions [39] and *RET* fusions [40]. Specific drugs are available for each of these alterations. Finally, *NTRK1-3* fusions are found with a low prevalence across different cancer types, including nonsquamous NSCLC [41], and are associated with the efficacy of tropomyosin receptor kinase (TRK) inhibitors (entrectinib and larotrectinib), which have received agnostic approval by the European Medicines Agency (EMA) and the Food and Drug Administration (FDA) [42,43].

In metastatic castration-resistant prostate adenocarcinoma, NGS should be used to detect *BRCA1/2* mutations or deletions, because when these alterations are present, poly (ADP-ribose) polymerase (PARP) inhibitors, such as olaparib, can improve patient outcomes [44]. *PTEN* testing should also be considered, since a recently published phase III randomized trial demonstrated that the combination of ipatasertib (an AKT inhibitor) and abiraterone significantly improved radiographic progression-free survival in patients with metastatic castration-resistant prostate adenocarcinoma characterized by a PTEN loss status by immunohistochemistry [45].

The use of NGS assays to determine the *BRCA1/2* mutational status is also recommended in ovarian carcinoma, in order to predict treatment response to PARP inhibitors [46,47].

In advanced cholangiocarcinoma, tumor multigene NGS could be used to detect IDH1 mutations, which are an actionable molecular target [48]. *FGFR2* fusions are also a predictive biomarker, since they are associated with the efficacy of pemigatinib, a selective fibroblast growth factor (FGF) receptor inhibitor [49]. In basket studies, patients with cholangiocarcinoma with microsatellite instability (MSI) and *NTRK* fusions noticed a benefit from therapies with immune checkpoint inhibitors (ICIs) and TRK inhibitors, respectively. Thus, a multigene NGS panel for this tumor may also include the evaluation of the *NTRK* and MSI status [50,51].

According to ESMO recommendations, NGS could also find an application in other tumor types apart from the four tumor types in which it is recommended. This is the case of metastatic colorectal adenocarcinoma [52]. In this cancer, hotspot *KRAS* and *NRAS* mutations and the *BRAF* V600E mutation are associated with resistance to anti-EGFR drugs (such as cetuximab) [53]. In *BRAF*-mutated tumors, a combination of cetuximab, binimetinib and encorafeinib (a BRAF inhibitor) can be used [54]. Another predictive biomarker in colorectal adenocarcinoma is the alteration of the DNA mismatch repair (MMR) [55,56], that can be identified through immunohistochemistry for MMR proteins (MLH1, PMS2, MSH2 and MSH6) and through the detection of MSI (which is a consequence of MMR deficiency) [52]. Hotspot mutations in *KRAS*, *NRAS* and *BRAF* and MMR deficiency (and/or MSI) can be detected through PCR and immunohistochemistry. In routine practice, the use of NGS is justified as an alternative to PCR if it does not generate additional costs [30]. When large NGS panels are used, *NTRK* fusions and *ERBB2* amplifications should also be assessed, because they can predict a response to TRK inhibitors and, according to recent prospective studies, anti-HER2 therapy, respectively [57,58].

Another application of NGS is the evaluation of the tumor mutation burden (TMB), defined as the mutation frequency in the tumor genome. This parameter has emerged as a predictive biomarker in several cancer types. In fact, a high TMB is associated with the response to ICIs, in particular to monoclonal antibodies directed against programmed death ligand-1 (PD-L1), such as pembrolizumab [59,60]. The gold standard method to determine TMB is WES, but an accurate estimation can be determined through the analysis of a defined gene panel by NGS [61]. According to ESMO recommendations, TMB should be determined in advanced cervical cancers, salivary gland cancers, thyroid cancers, neuroendocrine tumors and vulvar cancers, since in these tumors, a high TMB can give the patient access to anti-PD-L1 therapy [30].

Currently, the routine use of large NGS panels is not recommended in daily practice, because there is no evidence that they bring additional benefit to patients. In fact, large panels are unlikely to lead to the identification of additional actionable molecular targets. Furthermore, this strategy would entail high costs, also deriving from off-label drug use. For the same reasons, routine tumor multigene NGS is only recommended in a subset of cancer types, in which it can apport a proven benefit to the patients.

However, it is recommended for clinical research centers to implement multigene sequencing, also using large NGS panels and CGP, in order to identify patients eligible for clinical trials and to acquire new information on cancer biology and promote the development of new therapeutic strategies.

## 4. Preanalytical Issue in NGS

NGS can be performed on nucleic acid isolated from any source; however, the most widely used template in clinical practice is formalin-fixed paraffin-embedded (FFPE) tissue. Other possible sources of nucleic acid include frozen tissue and fluid samples (i.e., liquid biopsy), such as plasma, urine or effusion.

Preanalytical issues in molecular pathology start in the operating room: during surgery, blood vessels are clumped in order to prevent bleeding. This procedure determines a warm ischemia (so defined because it occurs at body temperature). After the excision, when the surgical piece is at room temperature or on ice, and until the completion fixation, the tissue undergoes cold ischemia [62]. A prolonged time interval between blood vessel ligation in the operating room and tissue fixation or freezing can provoke quantitative and qualitative alterations of nucleic acid, in particular RNA. In fact, ischemia may lead to the increased or reduced transcription of some genes, modifying RNA levels, even after a short interval (30 min or less) [63]. In addition, the extensive degradation of RNA due to endogenous enzymes can occur if there is a delay in tissue fixation or preservation [64,65].

Formalin fixation is the most critical step in the preanalytical phase. An adequate fixation time is of pivotal importance in order to preserve, as much as possible, the integrity of nucleic acids. A 12–24 h fixation in neutral buffered formalin is usually recommended to obtain a good sample preservation for a morphological evaluation. A shorter time may lead to incomplete fixation, which allows the enzymatic degradation of the tissue, leading to a suboptimal morphology, while a longer time may cause more extensive cross-linking and a more difficult extraction of DNA and RNA [1,66]. Formalin fixation can alter nucleic acids, determining strand breaks, base losses and cross-linking with other biomolecules; in addition, the fixation process also induces the deamination of cytosine and 5-methylcytosine, with the formation of uracil and thymine, respectively [67,68]. Multiple strand breaks result in the fragmentation of nucleic acids. In particular, RNA from FFPE samples can undergo extensive fragmentation, so that only sequences of approximately 100–200 nucleotides can be recognized and amplified through PCR [62,69]. Moreover, artifactual sequence changes that can result from the formalin-induced deamination of DNA and RNA bases may be erroneously interpreted as clinically relevant mutations [67]. In particular, a fixation time longer than 48 h seems to be associated with a significant increase in C:G > T:A mutations [70].

Decalcification is required for the processing of bone or other calcified tissues, and it can be carried out using strong acids (hydrochloric or nitric acid), weak acids (formic, picric or acetic acid) or chelating agents (EDTA). This process can further degrade nucleic acids to varying degrees, depending on the method used. EDTA and short-term formic acid-based decalcification allows for the detection of gene mutations, amplifications or even fusion transcripts, but they have the disadvantage of taking a longer time. On the other hand, strong acid decalcification is much faster but determines excessive DNA and RNA degradation and is, therefore, not suitable for molecular testing [71].

The long-term storage of FFPE tissue blocks is another preanalytical factor that can influence the quality of nucleic acids. In fact, the DNA and RNA extraction yield may decrease with increasing storage time [72]. It has been shown that after 4 to 6 years of storage of FFPE blocks, the amount of DNA measured by fluorimetry was reduced to 47%, and only 11% of DNA was amplifiable [73]. More recently, the researchers of the SCRUM-Japan GI-SCREEN Pathology Group studied the impact of the FFPE blocks storage period on the NGS success rates using the Oncomine Cancer Research Panel. They found that the success rate continuously decreased in accordance with the storage period and declined to 50% in 4 years [74]. These results highlight that we should improve strategies for the storage and preservation of FFPE tissue specimens. According to some authors, storage at lower temperatures, such as 4 °C, or freezing should be considered in prospective clinical studies [72]. However, such a strategy would increment storage costs and further studies are needed to confirm its validity.

### 4.1. Histologic Specimens

Histologic specimens can be derived from surgical resection or excision, or from a biopsy procedure. In the first case, the tumor is usually well represented, while in a biopsy sample, only a small amount of tumor tissue is sometimes present and it can be further limited by performing other ancillary investigations, such as special staining, immunohistochemistry and in situ hybridization. The total amount of tumor tissue is only one of the factors that can influence the DNA or RNA yield, a critical quality control step for NGS. Other factors include tumor cellularity and characteristics. Low-cellularity lesions, especially if small, require more unstained sections for nucleic acid extraction [75]. The tumor type is also an important variable: cystic, sclerotic, mucinous or necrotic areas may have a lower tumor cellularity and nucleic acid yield. The DNA input required for NGS depends on the platform used, the size of the gene panel and the target enrichment method. For example, the semiconductor-based Ion Personal Genome Machine (PGM) Sequencer allows for testing of small tumor samples using only a few nanograms of DNA [76], while technologies using the MiSeq System have a higher DNA input requirement [75].

The tumor fraction is another important parameter to consider. The tumor fraction requirement depends on the analytical sensitivity of the platform: the lower the limit of detection of the platform, the lower the tumor fraction necessary for the identification of a low-frequency variant. In order to ensure the selection of appropriate and representative tissue and to obtain an acceptable tumor fraction, pathologists can circle tumor-rich areas on stained sections, excluding non-neoplastic tissue, tumor necrosis and areas with an excessive inflammatory component. The selected area can then be dissected in unstained sections and sent to molecular testing [77].

In this context, the neoplastic cell content determination performed by the pathologist is crucial for biomarker testing. An external quality assessment program for metastatic colorectal cancer from the European Society of Pathology found an alarming interpathologist variability in the delineation of the tumor area, in the estimation of the neoplastic cell content and in the interpretation of the results. Further training and the standardization of practicing are necessary for minimizing preanalytical and postanalytical errors [78].

### 4.2. Cytologic Specimens

An NGS mutational analysis can be performed using cytologic specimens. In some cases, the cytologic specimen is the only sample available for diagnosis and molecular testing, especially in advanced disease and in patients with a low performance status who are not candidates to open biopsy. Cytology is a rapid, minimally invasive and generally well-tolerated procedure. Moreover, at many institutions, a rapid on-site evaluation (ROSE) helps ensure adequate samples are available for the diagnostic evaluation [79].

There are different types of cytologic preparations, such as direct smears, cytospin preparations, liquid-based cytology and FFPE cell blocks. Even if it can vary at different institutions, the procedure for obtaining a cell block is similar to typical surgical pathologic processing and it generally involves fixation and subsequent paraffin embedding. A significant advantage of cell blocks is that they allow the pathologist to select the material for the molecular analysis; the biggest disadvantage is the inability to assess them immediately for adequacy. Another disadvantage is that fixation and inclusion processes are associated with a deterioration of nucleic acid quality; moreover, during the section preparation, especially if the microtome is not meticulously cleaned and the knife is not regularly replaced, the cross-contamination of the material may occur [77]. On the other hand, direct smears, cytospin preparations and liquid-based cytologic preparations are not formalin-fixed and paraffin-embedded; thus, allowing the extraction of high-quality DNA and RNA, which is nearly impossible in traditional histology [75]. Liquid-based cytologic preparations share with cell blocks the disadvantage of not being immediately evaluable for adequacy; however, the reduction in background non-neoplastic material, such as blood, inflammatory cells and mucus, makes liquid-based cytology a valid cytologic preparation method for NGS [80,81].

An important issue with cytologic specimens is that NGS assays have generally been designated and validated only on FFPE biospecimens, and many molecular laboratories are not validated to test all the different types of cytologic preparations [82].

Among preanalytical issues in cytology based NGS, there are the cellularity of the specimen and the tumor fraction. The cellularity mainly depends on the quality of the procedure for obtaining the cytologic sample and on the characteristics of the tumor. For example, cystic, sclerotic or necrotic tumors often have a low cellularity, while tumors composed of poorly cohesive cells, such as melanoma or some neuroendocrine neoplasms, usually have a higher yield [69]. The tumor fraction also depends in part on the characteristics of the tumor, but it is also determined by the type of specimen. For example, fluid samples and endobronchial ultrasound-guided lymph node fine-needle aspirations (FNAs) often have a low tumor fraction, due to the presence of a large amount of non-neoplastic cells (such as mesothelial cells, histiocytes and lymphocytes).

The minimum cellular cutoff required for an NGS analysis depends on both the target capture and the platform used [83]. For example, Illumina NGS requires approximately 15,000 cells when following hybridization capture, whereas Ion Torrent NGS needs between 100 and 1000 cells [84,85]. Moreover, as already stated, even the DNA input ranges widely based on the platform used.

**Figure 1:**
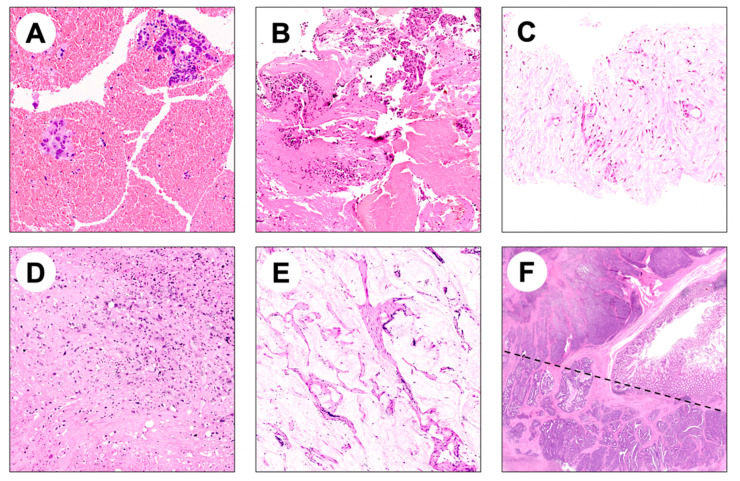
Representative images of potential pitfalls in NGS analysis of FFPE bioptic and surgical specimens. (**A**,**B**) Hematic material enclosing few adenocarcinoma glands; (**C**) biopsy specimen composed of fibrotic tissue enclosing rare adenocarcinoma glands; (**D**) scattered tumor cells surrounded by necrosis and fibrosis in a metastatic surgical resection specimen; (**E**) mucinous adenocarcinoma characterized by low cellularity and mucinous acellular component; (**F**) intratumor heterogeneity may hamper NGS analysis if both components are not considered (a morphologically heterogeneous colorectal adenocarcinoma composed by glandular and solid areas is shown).

**Figure 2:**
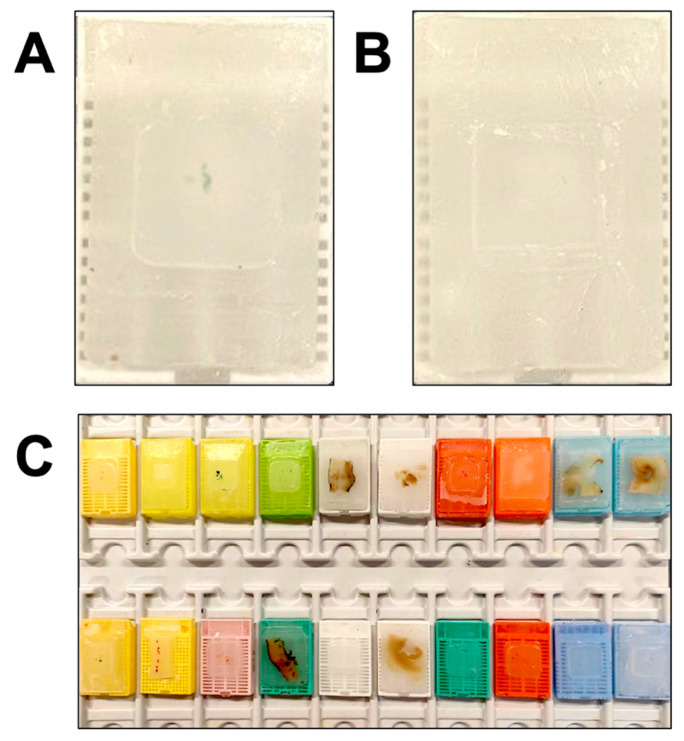
Representative images of FFPE tissue blocks inadequate for NGS analysis due to (**A**) scarcity of material (**B**) and absence of material following previous sectioning for diagnostic purposes. (**C**) A hub center for NGS-based molecular diagnostics receives different types of FFPE tissue specimens (i.e., biopsy, surgical resection and cytology specimens) obtained with different workflows and processes.

Representative images of histologic specimens and FFPE tissue blocks raising preanalytical issues for NGS-based molecular diagnostics are reported in Figure 1 and Figure 2, respectively.

## 5. Analytical Issue in NGS

The most common analytical confounders in NGS include C:G > T:A substitutions during amplification, resulting from the deamination of cytosine bases. Cytosine deamination is a frequent phenomenon in nature, and specific DNA repair systems exist to correct this alteration [86]. One of the two deamination mechanisms is the deamination of 5-methylcytosine, which generates thymine. This alteration can be repaired by the enzyme thymine-DNA glycosylase, but if the DNA is replicated before the error is corrected, a cytosine to thymine base substitution is generated. The other mechanism is the hydrolysis of cytosine into uracil. This alteration is corrected by the enzyme uracil N-glycosylase, which removes the uracil base generating an abasic site; subsequently, a cytosine is added to restore the original sequence [87]. Again, if the error is not fixed before replication, a U:A mutation is generated, which is converted into T:A during the subsequent round of synthesis. Deamination can have a biologic source, meaning that it can be intrinsic to the sample prior to isolation, but it can also be induced by formalin fixation [88], in this case representing a preanalytical issue. However, it has been demonstrated that cytosine deamination also represents an analytical problem, since it can occur during PCR, as a consequence of the heat from the denaturation phase of thermocycling [89].

Another important analytical issue that occurs when using multiplex-PCR-based NGS is amplicon mispriming, which can lead to the detection of false-positive mutations. During PCR, the primers recognize the 5′ and 3′ extremities of each amplicon. However, in some cases, another primer in the solution matches with a partially complementary internal region of the amplicon. This mismatch determines the generation of an amplification product that is shorter than the full-length amplicon and contains apparent mutations deriving from the primer mismatching in the middle of the true amplicon. Generally, false positives due to mispriming are easily identified through an bioinformatic analysis, because they occur in the same location, within the same amplicon and in reads that are shorter than full length [90].

## 6. RNA-Based NGS

Through NGS, it is possible to sequence RNA. As NGS technology uses DNA, this requires the reverse transcription of RNA into cDNA before sequencing. RNA-based NGS (often called RNA-seq) can provide information about the gene expression level, novel splice isoforms, transcript mutations and can also detect gene fusions [91,92]. In particular, in recent times, RNA-seq has increasingly been used to detect translocations or splicing alterations that can have a predictive value in cancer (for example, *ALK*, *ROS1*, *RET* and *NTRK* fusions and *MET* exon 14 skipping mutations in NSCLC) [36,37].

Translocations can occur anywhere in the genome, including exons, introns, intergenic regions and other noncoding sequences. Through DNA-based NGS, the detection of a gene fusion can be complicated by the presence of large intronic regions, which are often difficult to amplify and sequence [93,94]. Analyzing mature mRNA, RNA-based sequencing is not affected by the intron size [95]. Moreover, in contrast to an DNA-based analysis, which can indiscriminately detect every gene fusion, RNA-seq only detects fusions that undergo transcription; thus, having a potential role in cancer.

RNA-seq can provide better results than DNA-based testing also in the detection of point mutations, insertions or deletions that alter gene splicing. It has been demonstrated that RNA-based NGS can detect a significantly higher proportion of MET exon 14 skipping cases compared to DNA-based NGS [96].

The primary concern about RNA-seq is the labile nature of RNA, whose quality in FFPE tissue samples may be too poor for clinical testing, especially in older blocks [97]. As previously stated, RNA can easily undergo alteration and fragmentation during the preanalytical phase. Highly damaged RNA may consist largely of fragments that are too short to be informative [98]. For this reason, even if RNA-based approaches are becoming widespread in the research setting, they are less commonly used compared to DNA sequencing in clinical practice. In the future, further technological developments, such as the development of new RNA preservative reagents, extraction methods and RNA capture/hybridization protocols, may enable the use of RNA-seq in molecular diagnostics [99].

## 7. NGS in Epigenetics

Epigenetics is the study of changes in gene expression that do not involve alterations in the DNA sequence [100]. Examples of epigenetic mechanisms are DNA methylation and histone modifications. There is a growing number of studies on the use of NGS to investigate epigenetics in cancer [101,102,103].

DNA methylation consists of the transfer of a methyl group in position five of cytosine, forming 5-methylcytosine. This process mainly occurs in the CpG islands, located in the promoter regions of some genes. Alterations in DNA methylation are a common event in tumor cells. In particular, hypermethylation can repress the gene transcription of tumor suppressor genes. Hypomethylation can also promote oncogenesis by the activation of oncogenes, or increasing chromosome instability [104]. The DNA methylation status can be studied through NGS using a technique called bisulfite sequencing, in which DNA is treated with sodium bisulfite. This reagent converts cytosine residues to uracil, but leaves 5-methylcytosine residues unaffected; thus, allowing the distinction between methylated and nonmethylated regions during the sequencing process [105].

The structure of chromatin, which consists of a DNA–protein complex, can be regulated by various processes, including histone modifications (such us histone acetylation and deacetylation, or histone methylation and demethylation). These modifications can alter the tridimensional structure of chromatin and the interaction between DNA and nuclear proteins, such us transcription factors [106]. The DNA–protein interaction can be studied using ChIP-sequencing, also known as ChIP-seq. This method implies the cross-linking of proteins to DNA and then the conversion of DNA strands into short fragments. DNA fragments are then immunoprecipitated using bead-attached antibodies against the protein of interest. Finally, DNA is separated from the proteins and sequenced, in order to identify the sequence that binds the protein of interest [107].

## 8. Interpretation of DNA Sequencing Data

**Figure 3:**
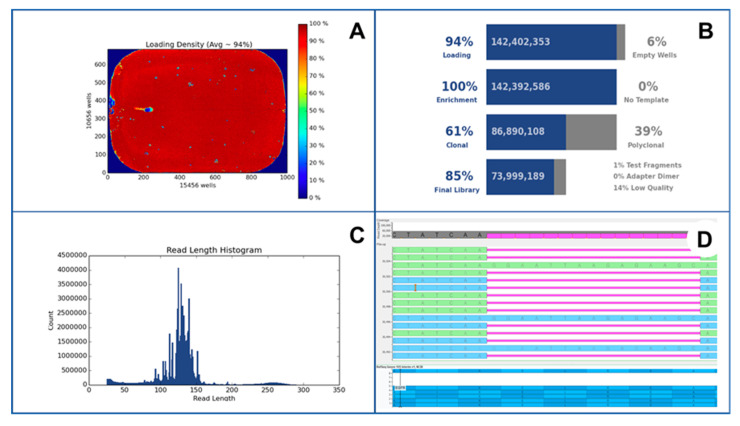
An exemplificative case of EGFR exon 19 deletion p.A746_A750 del detected by using NGS Ion Torrent S5 (Thermo Fisher Scientifics) platform. In this figure, loading density (**A**), technical quality parameters (**B**), read length histogram (**C**) and visual inspection of detected mutations with Golden Helix Genome Browse tool (**D**) were observed.

NGS produces a large amount of data, whose difficult interpretation remains an obstacle to the routine use of this technology. A crucial step in the NGS analysis is represented by data analysis. In this setting, bioinformatic pipelines play a crucial role in the NGS process. In particular, raw data inspection significantly impacts on the clinical administration of cancer patients. A plethora of different bioinformatic pipelines is currently available in the clinical setting. In this scenario, it is strongly recommended that a highly qualified and trained bioinformatician should optimize standardized guidelines in order to improve the quality of the NGS data analysis [108]. An example of data output from NGS sequencing is shown in Figure 3.

The first step is the conversion of the raw data produced by the sequencing instrument into base sequences, a process named “base-calling” [109]. This process generates sequences of letters, each corresponding to a specific nucleotide. A continuous string of letters represents a read, and every letter represents an estimate of the true nucleotide. A quality score can be assigned to each base-call reflecting the confidence that the true nucleotide has been correctly identified [25]. The reads are then aligned with the specific loci of a reference genome, available in online databases, a step known as reference mapping [110]. The next process, called variant calling, consists of identifying abnormalities in the DNA sequence. In this phase, the challenge is to separate true variants from background noise [111]. There are many bioinformatic programs specifically developed for variant calling that calculate the probability of each variant being a true variant, based on the known sequencing errors and polymorphism rate [25]. The ability of NGS to identify a specific abnormality in the DNA sequence largely depends on the type of alteration. Single-nucleotide variations are reliably detected alterations, while the detection of insertions, deletions and structural variants (such as translocations) is more challenging using DNA-based NGS, even if specific software has been designed to identify a structural variant by looking for the flanking regions of the reads [112].

One of the challenges in the use of NGS is the lack of standardization, not only of the platforms, devices and reagents, but also of the interpretation of sequencing data. It is desirable that shared guidelines are established to guarantee a standard of quality for NGS-based diagnostic services.

## 9. Conclusions

In the last decade, the increasing complexity of precision oncology, alongside major technological advances, has profoundly changed pathology practice. With the translation of NGS to the clinic, the pathologist has become a crucial figure in the oncologic therapeutic decision-making process and has acquired the responsibility of delivering a combined morpho-molecular characterization of neoplasms with diagnostic, prognostic and predictive value. To achieve this, there is a compelling need for pathologists to gain knowledge in molecular diagnostics, in order to avoid preanalytical and analytical errors, which may eventually hamper the delivery of the best treatment to the patient. The advent of NGS has also transformed both laboratory frameworks and the pathology working group, which now actively involves molecular biologists, laboratory technicians specialized in molecular diagnostics and bioinformaticians. The use of NGS in clinical practice also poses several challenges that should be addressed in the near future: the improvement of technical and regulatory infrastructures, the incorporation of bioinformatics in the diagnostic armamentarium, the clinically meaningful interpretation of the increasing body of molecular data and the promotion of genomic education among clinicians.

## Figures and Tables

**Table 1:** Comparison of different NGS platforms: Illumina MiSeq, Illumina HiSeq 2000, Ion Torrent PGM, PacBio SMRT and Oxford Nanopore MinION.

	Illumina MiSeq	Illumina HiSeq 2000	Ion Torrent PGM	PacBio SMRT	Oxford Nanopore MinION
Read Length	Up to 150 bases	Up to 150 bases	~200 bases	Average 1500 bases	13–20 kb
Paired-End	Yes	Yes	Yes	No	No
Reported Accuracy	Mostly >Q30	Mostly >Q30	Mostly Q20	<Q10	Mostly Q50
Observed Raw Error Rate	0.80%	0.26%	1.71%	12.86%	10.50%
Insert Size	Up to 700 bases	Up to 700 bases	Up to 250 bases	Up to 10 kb	Average of 331 bases
Run Time	27 h	11 days	2 h	2 h	72 h

## Data Availability

Data available upon request.
